# Supplementary material for: The acute effects of knee extension exercises with different contraction durations on the subsequent maximal knee extension torque among athletes with different strength levels
Source: PLoS One. 2022 Oct 27;17(10):e0267523. doi: 10.1371/journal.pone.0267523 (PMC9612496; doi:10.1371/journal.pone.0267523)
Supplement: Attachment — Submitted filename: Response to Reviewers.docx [file pone.0267523.s001.docx]

**Response to Editor**

***Comment 1****: Clarify whether the values in brackets after ICC are range of confidence intervals and at which level (e.g., 90%, 95%)*

**Response:** The values after ICC means a range of confidence intervals, and the level is 95%. Therefore, we have changed the following the text from (Line 225-227)

“The ICC values were 0.962 (0.897–0.989) and 0.998 (0.995–1.000) for voluntary knee extension peak torque and twitch peak torque, respectively.”

to

“The ICC values were 0.962 (95% confidence interval (CI): 0.897–0.989) and 0.998 (95% CI: 0.995–1.000) for voluntary knee extension peak torque and twitch peak torque, respectively.”

***Comment 2:*** *Please explain the advantages of the regression-discontinuity design used as opposed to a correlation between strength scores and PAP/PAPE achieved.*

**Response:** The primary objective of this study was to determine what is the contraction duration of the CA in which athletes with high and low muscle strength levels exhibit the highest PAPE, respectively. We believe that observing the correlation between strength scores and PAPE does not allow us to clarify the primary objective of this study. Furthermore, observing the changes in PAP/PAPE over time is also an important perspective in understanding the mechanism of PAPE. In order to clarify this point of view, we believe that it is effective to employ a regression-discontinuity design and observe the changes in PAP/PAPE over time after each CA is performed.

**Response to Dr Theodoros M. Bampouras (academic editor):**

We thank the publisher for considering our study for publication. We have revised the manuscript based on the editor's comments.

Journal Requirements:

1. Please ensure that your manuscript meets PLOS ONE's style requirements, including those for file naming. The PLOS ONE style templates can be found at 
<https://journals.plos.org/plosone/s/file?id=wjVg/PLOSOne_formatting_sample_main_body.pdf> and 
<https://journals.plos.org/plosone/s/file?id=ba62/PLOSOne_formatting_sample_title_authors_affiliations.pdf>

**Response:** We have revised the manuscript to conform to the format required by PLoS One.

2. Please provide additional details regarding participant consent. In the ethics statement in the Methods and online submission information, please ensure that you have specified (1) whether consent was informed and (2) what type you obtained (for instance, written or verbal, and if verbal, how it was documented and witnessed). If your study included minors, state whether you obtained consent from parents or guardians. If the need for consent was waived by the ethics committee, please include this information.

**Response:** In accordance with the editor's comment, we have added the text that we provided oral and written explanations to the participants and that we obtained their written consent (Line 112-114). In addition, because this study was a low-invasive experimental method, it was not necessary to obtain parental consent for the participation of minors under the approval of the Ethics Committee. Therefore, we have added a description of it to Line 117-119.

3. In your Data Availability statement, you have not specified where the minimal data set underlying the results described in your manuscript can be found. PLOS defines a study's minimal data set as the underlying data used to reach the conclusions drawn in the manuscript and any additional data required to replicate the reported study findings in their entirety. All PLOS journals require that the minimal data set be made fully available. For more information about our data policy, please see <http://journals.plos.org/plosone/s/data-availability>.

"Upon re-submitting your revised manuscript, please upload your study’s minimal underlying data set as either Supporting Information files or to a stable, public repository and include the relevant URLs, DOIs, or accession numbers within your revised cover letter. For a list of acceptable repositories, please see <http://journals.plos.org/plosone/s/data-availability#loc-recommended-repositories>. Any potentially identifying patient information must be fully anonymized.

Important: If there are ethical or legal restrictions to sharing your data publicly, please explain these restrictions in detail. Please see our guidelines for more information on what we consider unacceptable restrictions to publicly sharing data: <http://journals.plos.org/plosone/s/data-availability#loc-unacceptable-data-access-restrictions>. Note that it is not acceptable for the authors to be the sole named individuals responsible for ensuring data access.

We will update your Data Availability statement to reflect the information you provide in your cover letter.

The data on which the results of the present study are based has been uploaded to a public repository. Please check the DOI below.

Doi: [10.6084/m9.figshare.20078579](https://doi.org/10.6084/m9.figshare.20078579)

[Note: HTML markup is below. Please do not edit.]

**Response to Reviewer 1:**

Reviewer #1: General
*This is an interesting study which seeks to examine the optimal contraction duration for maximising voluntary performance and whether this varies depending on the strength level of an individual. There are some suggestions for improving the introduction and methods in particular and some questions that require consideration around the procedures and analyses.*

We wish to express our appreciation to the reviewers for their insightful comments on our paper. The comments have helped us significantly improve the paper.

Introduction
*General – there are a number of abbreviations introduced and used in this section which impact on its readability. Please consider whether all of these (e.g. SI and WI) are necessary.*

**Response:** As the reviewer pointed out, the introduction was difficult to understand due to unconventional abbreviations. Therefore, throughout the text, SI, WI, and P-MRLC have been changed to stronger individuals, weaker individuals, and phosphorylation of myosin regulatory light chains, respectively.

*General – this is a rather long (6 paragraphs) section in which the rationale for the investigation is built but if often theoretical in nature. Please consider highlighting the practical application(s) of the research questions that you pose for the reader to fully appreciate the need for the study.*

**Response:** We strongly agree with the reviewer on this point. Therefore, we have removed some of the rational for the investigation in the introduction and added the following description regarding the practical examples of PAPE. (Line 47-51)

In practice, coaches can utilize PAPE through high-intensity resistance exercises as CA to enhance the performance of subsequent plyometric exercises (i.e., complex training). Alternately, they can help enhance performance by employing CA immediately prior to the actual competitive performance (e.g., 100-m performance).

In addition, due to a lack of explanation of the practical applications of this research, the following text has been added to the final paragraph. (Line 101-102)

This hypothesis, if proven, will reaffirm the importance of individualizing strategies based on the level of muscle strength.

*Line 56 – please provide a supporting citation for this given the ‘factual’ statement.*
**Response:** In accordance with the reviewer's comment, we have added the supporting citation (Line 60).

Methods
*Line 114 – please refer the reader to table 1 for details of participant characteristics.*

**Response:** In accordance with the reviewer's comment, Table 1 was referred to Line 106.

*Line 115 – what is meant by regular resistance training (i.e. structured vs recreational, frequency)?*

**Response:** In accordance with the reviewer's comment, we have added the following text to the Methods (Line 106-108).

All participants had performed traditional resistance exercises, including high-intensity squats, approximately twice a week for at least six months.

*Line 128 – why was this method of normalising strength levels preferred over other options?*

**Response:** We wish to thank the reviewer for this comment. We believe that it is important to employ relative muscle strength index to separate the subjects' muscle strength levels in order to achieve the objectives of this study. This is because the adaptations that occur with high-intensity training (high fatigue resistance to high-intensity CA, high content of fast-twitch muscle fibers), which are important in this study, are better reflected in relative muscle strength than in absolute muscle strength. If absolute muscle strength were used as the classification index, we believe it would not adequately reflect these adaptations that can occur with high-intensity training, as the effect of body size would likely influence group classification.

*Line 131 – was 24 hours a sufficient recovery period considering the demands of the study?*

**Response:** We appreciate the reviewer's comment on this point. As the reviewer pointed out, sufficient rest between sessions is necessary in this study because the effects of fatigue may affect the results. However, all the exercises used in this study were concentric contractions, and previous studies have confirmed that the muscle strength loss that occurs after concentric contractions of the knee is fully recovered the next day^1)^. Therefore, it is expected that the minimum recovery period of 24 hours employed in this study was an adequate rest period.

1): Souron, R., Nosaka, K. & Jubeau, M. Changes in central and peripheral neuromuscular fatigue indices after concentric versus eccentric contractions of the knee extensors. Eur J Appl Physiol 118, 805–816 (2018). <https://doi.org/10.1007/s00421-018-3816-0>

*Line 135 – details of the CAs are provided (i.e. joint velocity) but it is not yet clear what activities were being used for the CA.*

**Response:** In accordance with the reviewer's comment, we have added the following the text regarding the details of the CAs employed in this study (Line 130-132).

“Isokinetic knee extension exercises were performed for dynamic CA, and the joint angular velocity of each CA was set at 60°/s.”

*Line 137-140 – the repeatability information may be better placed in the description of the protocols and not in the study design sub-section.*

**Response:** In accordance with the reviewer's comment, the repeatability information has been placed in the paragraph of the “Experiment session” (Line 223-227).

*Line 141 – elaborate on the sufficiency of 10 minutes rest given pre-testing activities that may have taken place. What is meant by ‘rest’ – stationary, seated?*

**Response:** In accordance with the reviewer's comment, we have changed the following the text from (Line 134-136)

“In all sessions, the participant was required to rest for 10 min after arriving at the laboratory to eliminate the effect of PAP induced by traveling to the laboratory”

to

“In all sessions, the participant was required to limit their activities such as walking for 10 min after arriving at the laboratory and remaining at rest to eliminate the effect of PAP induced while traveling to the laboratory”

In addition, we have added the following text to explain that 10-minute rest period is sufficient to eliminate PAP (Line 136-137)

“Given that PAP declines quasi-exponentially and is negligible after about 5 minutes [1,2], this rest period is sufficient to dissipate the effects of PAP”

*Line 143-149 – again, these details may be better placed in the description of the protocols and not in the study design sub-section.*

**Response:** In accordance with the reviewer's comment, we have moved the description of dynamometer settings to Line 146-149, and the description of analog-to-digital conversion to Line 162-165.

*Line 146 – how did ‘alignment’ take place?*

**Response:** In accordance with the reviewer's comment, we have added the following the text to Line 149-151.

“Based on a previous study by Miyamoto et al. [23] the knee and hip joint flexion angles were fixed at 90° and 80°, respectively”

*Line 149 – on what basis was a cut-off frequency of 12 Hz selected?*

**Response:** The cutoff frequency of 12 Hz was determined by our own preliminary experiments. Preliminary experiments confirmed that a cutoff frequency of 12 Hz eliminates noise due to motion and avoids excessive smoothing.

Therefore, we have added the following text to Line 166-168 regarding the determination of the cutoff frequency.

“This cutoff frequency was determined in our preliminary experiments, and we have confirmed that it avoids excessive smoothing and eliminates noise due to motion.”

*Line 158 – please justify your choice of instruction given the variability in peak torque and rate of force development that has been shown to occur with different instructions.*

**Response:** In accordance with the reviewer's comment, we have added the following the text (Line 152).

“This instruction is considered favorable for measuring maximal force and power [24,25].”

*Lines 234-249 – the statistical analysis needs to be explained in a way that is clearly related to the research questions. e.g. why were Pearson’s correlations used? Did the use of independent t-tests to examine difference between SI and WI not depend on the outcomes of the ANOVA considering the study design used?*

**Response:** We thank the reviewer for this comment. Certainly, the correlation between strength level and the degree of PAP is not a result that directly confirms the objective of this study. However, if differences were observed between stronger individuals and weaker individuals in the results of PAPE after performing the CA with the same contraction duration, we thought that the results of the correlation between strength level and the degree of PAP, as shown in Figure 2, could provide further insight into the cause of these differences. Furthermore, we believe that the use of independent t-tests to identify differences in muscle strength levels between stronger and weaker individuals leads to a strong indication that the results presented in Figures 4 and 5 are the results of groups with different muscle strength levels. This is important for the purpose of this study, which is to show the results of PAPE for athletes of different strength levels.

Results
*Line 254 – consider using ‘**independent’ t-test rather than ‘unpaired’*

**Response:** In accordance with the reviewer's comment, we have changed “unpaired” to “independent” (Line 239).

*Table 1 – height is not presented in metres. MVC/Weight is actually body mass (kg).*

**Response:** In accordance with the reviewer's comment, we have changed “m” to “cm” and changed “Weight” to “Body mass” (Table 1).

Discussion
*Line 357-359 – include supporting citations*

**Response:** In accordance with the reviewer's comment, references were cited in the relevant text (Line 368).

*Line 362 – reword for clarity*

**Response:** In accordance with the reviewer's comment, we have changed the following the text from (Line 373-374).

“We should note why SI was unable to improve the voluntary performance in the condition in which PAP was present in the 6-CA protocol (i.e., Post-10 s, Post-1 min, Fig 4A and Fig 5A)”

to

“Notably, the group of stronger individuals showed PAP at Post-10 s and Post-1 min of the 6-CA protocol (Figure 4A), but failed to exhibit PAPE during the same time (Figure 5A)”

*Line 385 – the presumption here is somewhat speculative. Please adapt that language to indicate that this may have been a plausible explanation.*

**Response:** In accordance with the reviewer's comment, we have revised the following text from (Line 396-399)

“Although their study did not state the years of resistance training experience, it can be presumed that the participants in their study had a longer resistance training history than those in the present study, which could explain the inconsistent results”

to

“Although their study did not state the number of years of resistance training, most of the participants in this study were college students who began resistance training in earnest during college years and most likely had fewer years of training than those in the study by Seitz et al. [14], which could explain the inconsistent results”

*Line 421 – are the authors confident that the findings do not reflect the study being underpowered given the limitations with sample size?*

**Response:** In accordance with the reviewer's comment, we believe that this study is not underpowered because we have confirmed that the power calculated from the sample size used in this study and the effect size obtained is above 0.8.

**Response to Reviewer 2:**

Reviewer #2: General comments:

*This study set out to compare the impacts of conditioning activity of varying durations on subsequent muscle performance, with additional comparisons between stronger and weaker individuals within the same cohort. Overall, this is a well-designed experiment with some interesting findings. I believe this study is certainly publishable, however, I do have several specific (minor) comments below that I feel could be addressed before publication of this manuscript. The comments below are generally line-specific, but I do have one general observation that the authors might want to consider:*

We appreciate you taking the time to offer us your comments and insights related to the paper. We found the reviewer’s feedback very constructive. The comments have helped us significantly improve the paper. We tried to be responsive to reviewer’s concerns.

*There are several “unconventional” abbreviations in this manuscript that might take the reader a while to become familiarised with. Because of this, I found parts of the introduction difficult to follow and felt the need to re-read sections a couple of times to understand the point. Perhaps the authors could consider removing one or two of the non-essential, and less frequently-used, abbreviations or even re-structure sentences/paragraphs in a way that make them more readable.

This, along with addressing other comments below, would likely improve the overall quality of the manuscript.*

**Response:** We wish to thank the reviewer for this comment. As the reviewer pointed out, the introduction was difficult to understand due to unconventional abbreviations and more theoretical content than necessary. Therefore, we have removed some abbreviations (i.e., SI→stronger individual, WI→weaker individual, P-MRLC→phosphorylation of myosin regulatory light chains). In addition, we have revised some theoretical content and reorganized the text to better convey the objectives of this study to the reader.

*Finally, I cannot see where to access the raw data for this project. Although I don’t think it impacted my ability to review the manuscript, I know it is a requirement of the journal.*

**Response:** The data on which the results of the present study are based has been uploaded to a public repository. Please check the DOI below.

Doi: [10.6084/m9.figshare.20078579](https://doi.org/10.6084/m9.figshare.20078579)

Specific comments:

Introduction
*Line 52-55: In which situations do coaches use CA? A couple of contextual examples here would be helpful, maybe just in parentheses.*

**Response:** We thank the reviewer for this comment, as supplementing reviewer’s points will help readers find the significance of this study in the field. Therefore, we have added the following text to the Introduction. (Line 47-51).

“In practice, coaches can utilize PAPE through high-intensity resistance exercises as CA to enhance the performance of subsequent plyometric exercises (i.e., complex training). Alternately, they can help enhance performance by employing CA immediately prior to the actual competitive performance (e.g., 100-m performance).”

In addition, we have added practical examples in parentheses for the areas the reviewer pointed out (Line 57-58).

*Line 95: Change to “…**resulting in a higher level of PAPE.”*
**Response:** In accordance with the reviewer's comment, we have changed the following text from (Line 85)

“resulting in a greater extent of PAPE”

to

“resulting in a higher level of PAPE”.

Methods
*Lines 146-149: At what sampling rate was the torque signals collected at?*

**Response:** In accordance with the reviewer's comment, we have added a description of sampling rate to Line 168.

*Line 149: What type of filter was used on these data, a low-pass* *Butterworth filter or something else? Also, what was the rationale behind the 12 Hz cut-off frequency? If this was based on previous dynamometry literature, then please cite necessary articles.*

**Response:** A low-pass Butterworth filter was used for the data (Line 165-166). The cutoff frequency of 12 Hz was determined by our own preliminary experiments, not based on previous literature. In our preliminary experiments, we confirmed that a cutoff frequency of 12 Hz eliminates noise caused by exercises and avoids excessive smoothing.

Therefore, we have added the following text to Line 166-168 regarding the determination of the cutoff frequency.

“This cutoff frequency was determined in our preliminary experiments, and we have confirmed that it avoids excessive smoothing and eliminates noise due to motion.”

*Lines 165-168: What is the reasoning behind this sample size? Why did the authors specifically take the strongest and weakest eight participants, and not more (let’s say, ten?)*

**Response:** We thank the reviewer for this comment. In order to achieve the objectives of this study, we believe it is necessary to clearly separate the differences in muscle strength levels between the stronger and weaker individuals group. However, the greater the number of subjects in each group, the more likely it is that the objectives of this study will not be achieved. Furthermore, if the number of subjects in each group is set to ten in this study, one subject will be classified into the stronger individuals group in terms of “absolute muscle strength” and classified into the weaker individuals group in terms of “relative muscle strength”, which makes the discussion somewhat difficult and may mislead the reader. For this reason, we chose eight subjects for each group, recognizing the fact that the sample size was somewhat small.

*Line 197: “posttest” should be hyphenated. Please change all occurrences to “post-test”.*

**Response:** In accordance with the reviewer's comment, we have changed “posttest” to “post-test” throughout the text.

*Lines 234-249: (Statistical analysis) Please cite Cohen (1988) study where relevant (lines 236 and 247).*
**Response:** In accordance with the reviewer's comment, we have cited the Cohen (1988) study (Line 242, Line 253).

Discussion
*Lines 343-346: Could the authors expand the mechanisms that might explain how muscle temperature increases could have led to the findings of the present study?*

**Response:** In accordance with the reviewer's comment, we have added the following text regarding the mechanism by which the increase in muscle temperature associated with the implementation of CA improves voluntary movement performance (Line 346-349).

“This is due to improved ATP turnover rate by facilitation of temperature-dependent myosin ATPase activity, which ultimately increases muscle shorting velocity [32,33]. In addition, the increase in muscle temperature is thought to decrease the viscous resistance of muscles and joints and increase the nerve conduction rate [34]”

*Line 353: typographical error – change to “**participants”.*

**Response:** This error has been corrected in accordance with the reviewer's comment (Line 363).

*Line 404: typographical error – change to “**participants”.*

**Response:** This error has been corrected in accordance with the reviewer's comment (Line 418).

*Lines 404-410: I wonder if, at this point, the authors could discuss the concept of “high-responders” and “low-responders” in PAP-type research. This might help explain some of differences observed between individuals with similar strength capabilities.*

**Response:** We appreciate the reviewer's concerns on this point. While we found the reviewers' comments valuable, we chose not to discuss the individual differences observed between the similar muscle strength levels in this study for the following two reasons. First, to the best of our knowledge, there are very few studies that discuss differences in the degree of PAPE among the similar muscle strength levels, and we believe that discussing this point based on the limited results of this study would lead to excessive speculation. Second, since explaining individual differences within similar muscle strength levels is somewhat different from the purpose of this study, we fear that by discussing this point, the purpose of this study may not be adequately conveyed. Therefore, while we consider the reviewer's comments to be an important point for future research on PAP-type, we have chosen to avoid discussing this point in this study.
