# Supplementary material for: The acute effects of knee extension exercises with different contraction durations on the subsequent maximal knee extension torque among athletes with different strength levels
Source: PLoS One. 2022 Oct 27;17(10):e0267523. doi: 10.1371/journal.pone.0267523 (PMC9612496; doi:10.1371/journal.pone.0267523)
Supplement: Attachment — Submitted filename: Response_to_Reviewers.docx [file pone.0267523.s002.docx]

**Response to Dr Theodoros M. Bampouras (academic editor):**

We thank the referees for their careful reading of the manuscript and the helpful suggestions. We feel the comments have helped us significantly improve the paper. In particular, we acknowledge their highly valuable comments regarding the comparison of the present study with the study by Seitz et al.

***Comment 1***

*Please justify the selection of the regression-discontinuity design over the correlation; in other words, why you believe that the correlation can not meet the study's objectives, especially as you have included later and suggested it strengthens the study. Information like this will help future researchers.*

**Response:** For example, if we were to correlate the PAPE value 4 min after one of the three types of CA with muscle strength level, we might be able to show that "athletes with higher muscle strength level have higher PAPE" but we would not be able to compare the results with those of other contraction durations, and we would not be able to clarify " what is the contraction duration of the CA in which athletes with high and low muscle strength levels exhibit the highest PAPE ", which was the primary objective of this research.
In addition, the timing of PAPE expression from CA implementation is important to understand the mechanism. For example, if PAPE is triggered around 1 min after CA, it is possible that PAP is involved as one of the mechanisms (i.e., the time period when PAP still remains). On the other hand, if PAPE is triggered 7 minutes after CA, the possibility of PAP being involved in the mechanism is extremely small (i.e., the time period when PAP has already disappeared). We believe that the correlation results are not suitable for understanding the mechanism of PAPE generation because it is not possible to correspond PAPE and PAP results for each time period.

***Comment 2***

*Please clarify in the Ethics statement that the minors that took part were able to, and gave, informed consent.*

**Response:** In accordance with the reviewer's comment, we have changed the following the text from (p 10, Lines 130-133)

“The participants were informed verbally and in writing about the procedures involved, and possible risks and benefits of the study, for which they provided written consent before the study commenced.”

to

“The participants were informed verbally and in writing about the procedures involved, and possible risks and benefits of the study, for which written informed consent was obtained from all study participants before the study commenced.”

***Comment 3***

*Please elaborate further on the resistance training programme - what is 'traditional' resistance exercises? In what part of the season were the players in? Information like this will help future researchers as well as literature comparisons.*

**Response:** We thank the reviewer for this comment. As the reviewer commented, the word “traditional” was confusing, so we have changed the word to “basic” (p10, Line 122-126).

In addition, we have added a description of the more detailed training program that the subjects were conducting and changed the following text from

“All participants had performed traditional resistance exercises, including high-intensity squats, approximately twice a week for at least six months.”

to

“All participants had performed basic resistance exercises, including high-intensity bench press and squats in a session of training, approximately twice a week for at least six months. In addition, the present study was conducted in the pre-season period, and power exercises such as power cleans were also introduced into the training program.”

***Comment 4***

*Please justify the normalisation method used, rather than why normalisation was used (e.g. https://pubmed.ncbi.nlm.nih.gov/12141882/ v* [*https://pubmed.ncbi.nlm.nih.gov/21187208/*](https://pubmed.ncbi.nlm.nih.gov/21187208/)*)*

**Response:** In accordance with the reviewer's comment, the following text has been added to Line 145-146 to justify the normalization method employed in this study.

“As body size affects the tested muscle strength, Jaric et al. [22] recommend normalization method that divides torque according to body mass.”

***Comment 5***

*Please provide the a-priori power calculation for the sample size (e.g. https://www.ncbi.nlm.nih.gov/pmc/articles/PMC7575068/). The use of post-hoc power analysis is incorrect, which has been previously discussed in various fields (e.g. https://gpsych.bmj.com/content/32/4/e100069, https://journals.lww.com/annalsofsurgery/Citation/2019/01000/Post_Hoc_Power_Calculation__Observing_the_Expected.48.aspx) and can result in misleading interpretation (e.g. https://pubmed.ncbi.nlm.nih.gov/32843199/,* [*http://daniellakens.blogspot.com/2014/12/observed-power-and-what-to-do-if-your.html*](http://daniellakens.blogspot.com/2014/12/observed-power-and-what-to-do-if-your.html)*).*

**Response:** We thank the Reviewer for this comment. In accordance with the reviewer's comment, we determined the sample size based on a-priori power calculation (medium effect size 0.25, an alpha level of 0.05, a power level of 0.8, and three groups), and the sample size was determined to be at least 8. Therefore, the following text has been added to p 14, Line 189-193:

“The G*Power software version 3.1 was used to determine the sample size of the stronger or weaker group. The following parameters were selected: medium effect size (f = 0.25), an alpha level of .05, a power level of 0.8. The sample size for both the stronger and weaker groups was determined to be at least 8.

Furthermore, the limitation on sample size in the discussion has been removed because it did not seem to fit the revised context. (p 36)

***Comment 6***

*Please provide some information regarding the preliminary experiments (e.g. mode, resemblance / identical to the present study).*

**Response:** In accordance with the reviewer's comment, we have provided a brief description of the preliminary experiment by adding the following text (p 15, Line 198-200):

“Specifically, it was determined by confirming that 3 standard deviation of the noise was no greater than the threshold of 0.5 N for the onset of contraction of isometric knee extension [27].”

***Comment 7***

*Please report height in m (I suspect there some confusion with the reviewer's initial statement re this aspect).*

**Response:** In accordance with the reviewer's comment, we have changed “cm” to “m”. (p 23)

***Comment 8***

*Please revisit the explanation regarding comparison between the present and Seitz et al study. Although the training experience is indeed not provided, the average knee extension MVC torque in the present study appears to be considerably higher than the Seitz et al study. Give the premise of the present study is that stronger people have higher PAP response, the training experience not resulting in increased torque seems irrelevant to the discussion.*

**Response:** We strongly appreciate the Reviewer's comment on this point. As the reviewer commented, it certainly appears that the participants in the present study may be stronger than the participants in Seitz et al.’s study. Thus, it is possible that our original speculation was incorrect.
The reason for this discrepancy in results is not clear, but other studies have reported large differences in potentiation effects between subjects, even at similar muscle strength levels. Therefore, it is possible that factors other than muscle strength caused the discrepancy in results between experiments, but it is unclear what these factors might be.
The discrepancies observed in the present study need to be examined in more detail in the future. Therefore, we have added the following the text (Line 456-462):

“Several previous studies have shown that the degree of potentiation effect of CA varies among individuals, even among subjects with similar muscle strength levels [41,42], but the factors that contribute to this difference have not been fully elucidated even today. Therefore, the reasons for the discrepancy between the results of the present study and those of Seitz et al. [14] remain unclear. Future studies are needed to elucidate in more detail the mechanisms involved in the individual differences in the degree of PAPE.”

**Response to Reviewer 2:**

Reviewer #2:

I would like to thank the authors for their careful responses to the comments from the Editor, my fellow reviewer, and myself. I believe the changes that have been made have improved the manuscript overall. Regarding my final initial comment about inter-individual differences, the authors’ decision not to act on this is fine; I agree with their justification. However, I do have two queries regarding their responses:

Again, we express our strong appreciation to the reviewers for their insightful comments on our paper. We have incorporated changes that reflect the detailed suggestions you have graciously provided. We also hope that our edits and the responses we provide below satisfactorily address all the issues and concerns you have noted.

*1) Regarding the choice of cut-off frequency used for filtering, this justification is insufficient. Confirming that a 12 Hz low-pass Butterworth filter “avoids excessive smoothing and eliminates noise due to motion” is vague and appears subjective. When you say that this was determined during preliminary experiments, do you mean you performed a residual analysis on pilot data? If so, please specify this. If not, and it was actually based on visual inspection of the data, then I think some explanation around what one defines as “excessive smoothing” is needed.*

**Response:** We appreciate the Reviewer's comment on this point. The 12 Hz determined as the cutoff frequency was based on visual inspection of the data. Specifically, we chose 12 Hz so that 3 standard deviation (SD) of the noise was no greater than the threshold of 0.5 N for contraction onset. The 3SD argument is advocated in a 2016 paper from Maffiuletti et al^1^. We also believe that this value is reasonable given the mode of exercise used in the present study. The isometric knee extension and isokinetic knee extension with an angular velocity of 180° take about 250-400 ms and 250 ms to reach maximal muscle force from the contraction onset, respectively. 1/0.25 = 4 Hz, therefore, we need to set a sample frequency of 8 Hz to measure 4 Hz (Nyquist frequency), and we added 50% to lose as little data as possible. That makes 12 Hz. That can be our definition of excessive smoothing.

Therefore, we have changed the following the text from (Line 196-200)

“This cutoff frequency was determined in our preliminary experiments, and we have confirmed that it avoids excessive smoothing and eliminates noise due to motion”

to

“This cutoff frequency was determined based on visual inspection in our preliminary experiments. Specifically, it was determined by confirming that 3 standard deviation of the noise was no greater than the threshold of 0.5 N for the contraction onset of isometric knee extension [27].”

1. Maffiuletti NA, Aagaard P, Blazevich AJ, Folland J, Tillin N, Duchateau J. Rate of force development: physiological and methodological considerations. Eur J Appl Physiol. 2016;116(6):1091-1116. doi:10.1007/s00421-016-3346-6
2. Aagaard P. Training-induced changes in neural function. Exerc Sport Sci Rev. 2003;31(2):61-67. doi:10.1097/00003677-200304000-00002
3. Oliveira AS, Corvino RB, Gonçalves M, Caputo F, Denadai BS. Effects of a single habituation session on neuromuscular isokinetic profile at different movement velocities. Eur J Appl Physiol. 2010;110(6):1127-1133. doi:10.1007/s00421-010-1599-z

*2) In response to my comment about sample size, the authors stated that “the greater the number of subjects in each group, the more likely it is that the objectives of this study will not be achieved”. Does this mean that you chose this sample size to allow you to confidently compare stronger and weaker individuals, or you chose this sample size to obtain the results you expected to before you conducted your experiments? It’s not clear to me, and this obviously has large implications for this point.*

**Response:** We thank the reviewer for this comment. The sample sizes for the stronger and weaker individuals selected for this study were determined prior to conducting the experiment in order to obtain our expected results. We believe that a sample size of 8 for each of the stronger and weaker individuals from the 22 participants in this study is preferable in terms of maintaining power while clearly separating muscle strength levels.

In addition, the following statement regarding the determination of the sample size for stronger or weaker individuals using the G*Power software version 3.1 has been added to Line 189-193

“The G*Power software version 3.1 was used to determine the sample size of the stronger or weaker group. The following parameters were selected: medium effect size (f = 0.25), an alpha level of .05, a power level of 0.8. The sample size for both the stronger and weaker groups was determined to be at least 8.”

Furthermore, the limitation on sample size in the discussion has been removed because it did not seem to fit the revised context (p 36).

Again, thank you for giving us the opportunity to strengthen our manuscript with your valuable comments and queries. We have worked hard to incorporate your feedback and hope that these revisions persuade you to accept our submission.
